# Brain and knee joint degeneration following anterior cruciate ligament injury in a mouse model of Alzheimer’s disease

**DOI:** 10.1093/jbmrpl/ziag085

**Published:** 2026-05-07

**Authors:** Wei Yao, Barton L Wise, Yu-Yang Lin, Ming Fan, Shanxiu Xu, I-Shin Ju, Jonathan I Berg, Jill L Silverman, Gabriela G Loots, Blaine A Christiansen

**Affiliations:** Department of Internal Medicine–Rheumatology, University of California Davis Health, Sacramento, CA 95817, United States; Department of Internal Medicine–Rheumatology, University of California Davis Health, Sacramento, CA 95817, United States; Department of Orthopedic Surgery, University of California Davis Health, Sacramento, CA 95817, United States; Department of Orthopedic Surgery, University of California Davis Health, Sacramento, CA 95817, United States; Department of Radiation Oncology, University of California Davis Health, Sacramento, CA 95817, United States; Department of Radiation Oncology, University of California Davis Health, Sacramento, CA 95817, United States; Department of Orthopedic Surgery, University of California Davis Health, Sacramento, CA 95817, United States; Department of Radiation Oncology, University of California Davis Health, Sacramento, CA 95817, United States; Department of Psychiatry and Behavioral Sciences, University of California Davis Health, Sacramento, CA 95817, United States; Department of Orthopedic Surgery, University of California Davis Health, Sacramento, CA 95817, United States; Department of Orthopedic Surgery, University of California Davis Health, Sacramento, CA 95817, United States

**Keywords:** osteoarthritis, behavior, joint degeneration, dementia, cognitive function, Alzheimer’s disease

## Abstract

Alzheimer’s disease (AD) and osteoarthritis (OA) are 2 of the most common health conditions affecting the elderly. Recent studies in mice and humans have described an association between OA and AD, with prevalent OA increasing the risk of developing AD-like cognitive declines. In this study, we investigated brain and knee joint degeneration and neurocognitive dysfunction in a mouse model of AD (APP/PS1 double transgenic mice) in the presence or absence of OA induced by non-invasive ACL rupture. We hypothesized that OA would be associated with increased beta amyloid (Aβ) and phosphorylated tau (pTau) in the injured joint and brain and would be associated with accelerated cognitive decline in APP/PS1-Tg mice. Non-invasive knee injury was performed at 7 mo of age in male and female APP/PS1-Tg mice and non-transgenic (WT) littermates. Neurocognitive tests, including open field, novel object recognition, and Morris water maze, were performed 6 wk post-injury. Knees were also assessed at 6 wk post-injury to quantify OA, and Aβ, Tau, and pTau levels in the brain and knee joints. We found that female APP/PS1-OA mice had significantly more osteophyte formation than WT-OA mice, and significantly greater Aβ and pTau levels in the brain and knee joints than in WT or APP/PS1-Sham mice. These results provide insights about the pathoetiology of AD and OA and suggest possible common mechanisms connecting OA-associated inflammation to cognitive function. Identifying novel mechanisms of crosstalk between these 2 conditions could improve clinical care and quality of life for at-risk patient populations.

## Introduction

Alzheimer’s disease (AD) and osteoarthritis (OA) are 2 of the most common health conditions affecting the elderly. One in 10 people aged 65 yr and older either have or will develop some form of dementia most often diagnosed as AD, and 10% of men and 18% of women over the age of 60 have OA.^1^ As the population of Americans aged 65 and older is projected to nearly double from 48 to 88 million by the year 2050, there will be an ever-growing burden on the healthcare system as considerably more individuals will be at high risk for both AD and OA.

Though not yet examined mechanistically, several recent human^2^^,^^3^ and mouse^4^^,^^5^ studies have revealed an association between OA and AD that warrants further investigation. Knee OA in humans has been found to be associated with amyloid deposits in the joints.^6^ A retrospective population-based 4-yr cohort study investigated the risk of dementia in OA patients using the Taiwan Longitudinal Health Insurance Database 2005.^7^ They found an adjusted hazard ratio for dementia of 1.25 (95% CI, 1.10-1.43, *p* < .001) for patients with OA, indicating that OA is an independent risk factor for dementia, though not specifically AD.^7^ A different analysis from the same group found a high prevalence of OA (38%) among incident AD cases, but this association disappeared after adjusting for age and sex, suggesting that the demographic profiles for AD and OA overlap considerably, possibly because of similar etiologies.^2^ Altogether, these observations suggest that there may be a common mechanism linking aging with both AD and OA.^8^ These studies introduce evidence that OA is associated with an increased likelihood of coincident AD and associated changes in the brain and raise the hypothesis that OA either exacerbates or triggers cognitive decline de novo.

The deposition of beta-amyloid protein (Aβ) outside neurons and the accumulation of phosphorylated Tau (pTau) inside neurons are characteristic changes associated with AD.^9^ Interestingly, Aβ has also been found in the joint capsules of osteoarthritic hips and there are suggestions of an association with chronic inflammation.^10^^,^^11^ Additionally, AD patients with OA had faster Aβ and pTau accumulation in their primary motor and somatosensory brain regions,^12^ supporting the hypothesis that chronic systemic inflammation precedes both AD and OA. Beta-amyloid deposition may also be a characteristic histopathological feature in advanced knee OA: aging induces amyloidogenic proteins and transthyretin formation in the joints in elderly patients with knee OA, whereas apolipoprotein A-I formation may be inversely correlated with age.^6^^,^^13^ Chondrocytes express Tau protein,^14^ and mice developed OA in a mouse model of AD that overexpresses a human form of Tau.^15^

APP/PS1 double transgenic mice express a chimeric mouse/human amyloid precursor protein (APP) and a mutant presenilin 1 (PS1), 2 mutations that are associated with early-onset AD, amyloid plaque formation, and premature aging.^16^ Beta-amyloid Aβ deposits in the brains of these mice are present by 6-7 mo of age, while cognitive deficits are observed by 9 mo of age.^16–18^ Therefore, these mice represent an established animal model for AD. This study aimed to determine whether crosstalk between the musculoskeletal system and the brain occurs during OA and/or AD development. We subjected APP/PS1 double transgenic mice and non-transgenic littermates to tibial compression-induced anterior cruciate ligament (ACL) rupture and evaluated their neurocognitive behavior and learning after post-traumatic osteoarthritis (PTOA) had developed. We hypothesized that OA-associated inflammation would drive earlier onset and faster progression of cognitive decline in a mouse model of AD. Investigating the long-suspected but incompletely understood link between these 2 common and debilitating diseases of aging could provide novel insights for understanding the pathoetiology of both AD and OA.

## Materials and methods

### Experimental design

Both APP/PS1 double transgenic mice (APP/PS1-Tg) and their non-transgenic WT littermate cohorts (C57BL/6J background) were obtained from the Jackson Laboratory (https://www.jax.org/strain/005864) and bred in house till they were 7 mo of age. Non-invasive knee injury (mechanically-induced ACL rupture)^19^ was performed at 7 mo of age in both female (F) and male (M) APP/PS1-Tg and WT mice. Cognitive and behavioral tests were performed 6 wk post-injury, a time point at which severe OA has developed in the injured joint.^20^^,^^21^ All mice were euthanized 6 wk post-injury. All animal procedures were approved by UC Davis Institutional Animal Care and Use Committee. A total of 90 mice were used for these procedures, with 7-14 mice/sex/strain/injury.

### Tibial compression overload ACL rupture (ACL-R)

Non-invasive knee joint injury (ACL rupture) was induced in mice as previously described.^19^^,^^22^ Mice were anesthetized, and the right lower leg was subjected to a single load of tibial compression (ElectroForce 3200, TA Instruments) at 1 mm/s until injury (~10-12 N). We have previously shown that this loading method induces ACL rupture and progresses to moderate-severe OA within 4-6 wk post-injury, including loss of epiphyseal trabecular bone, thickening of subchondral cortical bone, erosion of articular cartilage, synovitis, and osteophyte formation.

Sham injured mice were anesthetized, and the right lower legs were subjected to a single load of tibial compression at 1 mm/s as described above to a target load below the knee injury threshold (~8 N). Force/displacement plots and manual palpation were used to confirm that no ACL injury occurred.

### Micro-CT analysis of epiphyseal trabecular bone and osteophyte formation

Knees were imaged using micro-CT (SCANCO μCT 35) to determine microstructure of epiphyseal trabecular bone of the distal femur and to quantify mineralized osteophyte volume. Scans were performed according to rodent bone structure analysis guidelines, with X-ray tube potential = 55 kVp, intensity = 114 μA, 10 μm isotropic nominal voxel size, and integration time of 900 ms.^23^ Trabecular bone was manually contoured in the distal femoral epiphysis, and included all trabecular bone enclosed by the growth plate. Mineralized osteophyte volume was determined as previously described.^20^^,^^24^ ROI contours were drawn around the patella, fabellae, and meniscal mineralized tissues, as well as heterotopic mineralized tissue attached to the distal femur and proximal tibia. Contralateral limb patella, fabellae, and meniscal mineralized tissue were also measured. Total mineralized osteophyte volume was calculated as the difference in bone volume between injured and contralateral limbs.

### Histological evaluation of knee joints

Knee joints were fixed in 4% paraformaldehyde for >48 h, then preserved in 70% ethanol. Joints were decalcified in 0.5 M ethylenediaminetetraacetic acid (EDTA), embedded in paraffin, sectioned in the sagittal plane (4 μm thick sections), and stained with H&E, or Safranin-O and fast green. Three sections from the medial articulation were selected for analysis for each mouse.^25^ Articular cartilage degradation (OARSI scoring) was performed using the scale described by Glasson et al.^25^ with grade 0-6, representing normal cartilage (Grade 0), loss of cartilage staining without obvious fibrillation (Grade 0.5), minor fibrillation (Grade 1), or fibrillation and/or volumetric loss of cartilage from <25% (Grade 2) to >75% (Grade 6).

Osteoarthritis Stage scoring was adapted from Pritzker et al.,^26^ with grading based on the percent involvement of surface area, and volume of the articulation, with a score of 0 = no OA present, 1 = <10% joint involvement, 2 = 10%-25% involvement, 3 = 25%-50% involvement, and 4 = >50% involvement. Osteoarthritis Stage score for each sample was calculated as a sum of the tibia OA Stage score and the femur OA Stage score for that sample (maximum total score of 8). Composite Score for each knee joint was calculated as the product of the tibia OARSI score and tibia OA Stage score plus the product of the femur OARSI score and OA Stage score. Osteophyte score was based on the size and extent of osteophytes encompassing the joint, with a score of 0 = no osteophytes present, 1 = osteophytes encompassing <25% of the joint, 2 = osteophytes encompassing 25%-50% of the joint, and 3 = osteophytes encompassing >50% of the joint.

### ELISA analysis of Aβ, Tau, and pTau levels in brain tissue and knee joints

Mouse β-Amyloid 1-42 ELISA Kit (Cat# KMB3441, Invitrogen) was used to determine the levels of Aβ42 in brain or knee samples from WT and APP/PS1-Tg mice. Briefly, knee samples were homogenized using stainless-steel beads and Bullet Blender Gold (BB24-AU, Next Advance Inc.) in 1.5 mL RINO screw-cap microcentrifuge tubes at 15 000 rpm for 20 min with the presence of homogenization buffer (5 M guanidine-HCl in 50 mM Tris, pH 8.0). Brain samples were pulverized thoroughly with a hand-held tissue homogenizer in the presence of homogenization buffer and sonicated. The homogenates were then diluted 10-fold with cold PBS with protease inhibitor cocktail and centrifuged at 3000 rpm for 20 min at 4 °C. The supernatant was carefully transferred to a new Eppendorf tube and protein concentration was normalized by BCA Protein Assay Kit.

Pre-diluted samples and standards were added to the wells and incubated for 2 h at room temperature. Then, the wells were washed 4 times with the washing solution provided in the kit. After washing, 100 μL of Ms Aβ42 Detection Antibody was added to the wells and incubated for 1 h at room temperature. The wells were again washed 4 times and incubated with Anti-Rabbit IgG HRP for another 30 min at room temperature. 100 μL of stabilized chromogen was added to each well including the blank well after washing 4 times and incubated for 30 min at room temperature in the dark. The reaction was stopped by adding equal volume of stop solution to each well. The absorbance was recorded using the microplate reader (Spectra Max M2e, Molecular Devices Co.) at 450 nm. Curve-fitting was obtained by SoftMax V5. Aβ1-42 was measured according to the manufacturer’s instructions and the absorbance was recorded using the microplate reader (Spectra Max M2e, Molecular Devices Co.) at 450 nm. Curve-fitting was obtained by SoftMax V5.

### Western blot analysis of Aβ, Tau, and pTau levels in brain tissue and knee joints

Brain samples from WT or APP/PS1-Tg mice with or without knee injury were thawed in 1X RIPA buffer (pH 7.4) in the presence of protease inhibitor cocktail (catalog# 11836170001, Millipore Sigma) and phenylmethylsulfonyl fluoride (PMSF). Brains were homogenized by hand-held tissue homogenizer, sonicated, and centrifuged at 12 000 rpm for 15 min at 4 °C. Supernatant was transferred to a new tube and protein concentration was determined by the BCA assay. Equal aliquots of protein were run in SDS-PAGE and transferred to PVDF membrane. After 1-h blocking with 5% non-fat milk in TBST, the membranes were incubated with primary antibody overnight at 4 °C. The following day, after secondary antibody conjugation, the membranes were visualized by KwikQuant Imager using ECL detection kit (Biosciences, Cat R1100). The primary antibodies against Tau (Cat# ab254256), pTau 181 (Cat# ab75679), pTau 217 (Cat# 44744), Aβ1-40 (Cat# PA3-16760), and Aβ1-42 (Cat#44-344) were purchased from (Abcam); β-actin (Cat#A5441) was purchased from Millipore Sigma.

### Congo red and Thioflavin S staining of the brain and knee joints

Congo red and Thioflavin S are the 2 major histological stains used to detect any form of amyloid. Brain and knee samples were fixed in 4% paraformaldehyde in 0.1 M sodium/potassium phosphate buffer. The samples were sent to UC Davis Pathology Core for embedding in paraffin, after which they were sectioned, stained with Congo Red (Sigma) and Thioflavin S (Sigma), and semi-quantitatively graded as previously described.^27^^,^^28^

### Neurobehavioral tests

The cognitive tests were performed at 6 wk post-injury on all APP/PS1-Tg and littermate WT mice under uniform conditions to provide a comprehensive evaluation of cognition covering abilities for exploration, locomotion, recognition memory, spatial navigation, aversive learning, and anxiety,^29^ most of which are impaired in aged or APP/PS1-Tg mice.^30–36^ All functional and behavioral tests were performed in the UC Davis MIND Institute Intellectual and Developmental Disabilities Research Center (IIDDRC) Mouse Behavior Core.

#### Open field

Each mouse was transferred to a novel open field enclosure (18″ × 18″) for 30 min at 30 lx. An infrared laser grid was used to trace animal movement during each 30 min test. The open field test was used to measure voluntary locomotive activity and general explorative behavior. Ethovision (Noldus) software was used to record sessions and analyze the total distance traveled and the average velocity of each animal during the session.

#### Novel object recognition

The novel object recognition (NOR) test was conducted as previously described,^37^^,^^38^ and consisted of 3 sessions: a 30-min exposure to the open field arena, a 10-min familiarization session consisting of 2 identical objects, and a 5-min recognition test consisting of one clean familiar object and 1 clean novel object. Object investigation was defined as time spent sniffing the object when the nose was oriented toward the object and the nose-object distance was 2 cm or less. Recognition memory was defined as spending substantially more time sniffing the novel object than the familiar object. The total time spent sniffing both objects was used as a measure of general exploration.

#### Spontaneous alternation

Y Maze Spontaneous Alternation is a behavioral test for measuring the willingness of rodents to explore new environments. Testing was performed in a Y-shaped maze with three white, opaque plastic arms at a 120° angle from each other. After introduction to the center of the maze, the mice were allowed to freely explore the three arms. Over the course of multiple arm entries, the subject should show a tendency to enter a less recently visited arm. The number of arm entries and the number of triads were recorded to calculate the percentage of alternation. An entry was considered to have occurred when all four limbs were within the arm.

#### Morris water maze

Spatial learning and memory were evaluated in a 120 cm diameter circular pool using Ethovision video tracking. Acquisition training consists of 4 trials a day until the control group reached criterion. Latency to reach the hidden platform was the independent variable using a criterion of 15 s or less as the definition of a successful acquisition of the hidden platform location. Swim speed was measured as a control for motor abilities. Probe trial confirmation of learning consisted of a 60 s trial with the platform removed. Probe trials were performed 3 h post-acquisition day and again 24 h later to assess long-term memory.

### Statistical analysis

Data were analyzed with 3-way analysis of variance (ANOVA) with sex, genetic strain, and injury status as factors to determine main effects and interactions. Post-hoc analyses were performed with Tukey-Kramer. GraphPad Prism (GraphPad Prism version 9, GraphPad Software) was used to analyze and plot the data, and significance was defined as *p* < .05.

## Results

### Female APP/PS1-Tg mice exhibited greater osteophyte formation following knee injury

**Figure 1:**
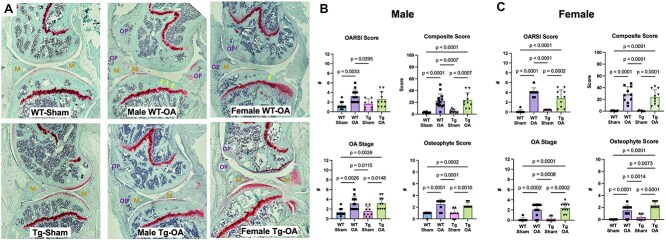
Knee joint pathology. (A) Representative histological images from Sham WT and TG (male) mice, and female and male OA WT and Tg mice. (B and C) Grading of articular cartilage degradation (OARSI Score), OA stage, osteophyte score, and composite score showed more severe joint degeneration in OA groups compared to Sham groups as expected. In female mice, significantly more osteophyte formation was observed in injured APP/PS1-Tg mice relative to WT-OA mice. No other significant differences were observed between Tg and WT mice. OP = osteophytes, M = meniscus, and Arrows = articular cartilage degeneration.

In female mice, knee injuries induced similar OA-like changes in both WT and APP/PS1-Tg mice with respect to OA grading, knee OA stage, and composite score. However, injured female APP/PS1-Tg mice had a significantly greater mean osteophyte score assessed on histological sections than injured WT mice (Figure 1). In contrast, knee injuries induced similar OA-like changes in both the WT and APP/PS1-Tg male mice, including similar osteophyte formation between APP/PS1-Tg mice and WT mice.

**Figure 2:**
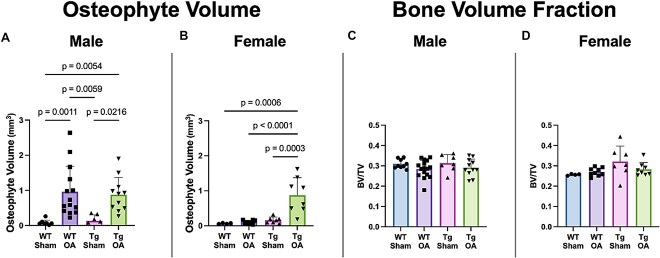
Micro-CT (μCT) results for mineralized osteophyte volume and epiphyseal trabecular bone microstructure. (A and B) Greater mineralized osteophyte volume was observed in both male OA groups compared to the male Sham groups. Greater osteophyte volume was also observed in female Tg-OA mice than in the other experimental groups. (C and D) There were no significant differences in epiphyseal trabecular bone volume fraction (BV/TV) or other microstructural outcomes based on sex, ACL injury, or genetic strain.

Micro-CT analysis of mineralized osteophyte volume further confirmed the findings from the whole-joint histological analysis, with significantly greater osteophyte volume in injured female APP/PS1-Tg mice than in female WT mice (Figure 2). In contrast, there were no differences in mineralized osteophyte volume between injured male APP/PS1-Tg mice and male WT mice. No significant differences were observed in distal femoral epiphysis trabecular bone microstructure between male or female APP/PS1-Tg or WT mice.

### Deposition of β-amyloid and activation of pTau in the brain was observed in APP/PS1-Tg mice and differed by sex

**Figure 3:**
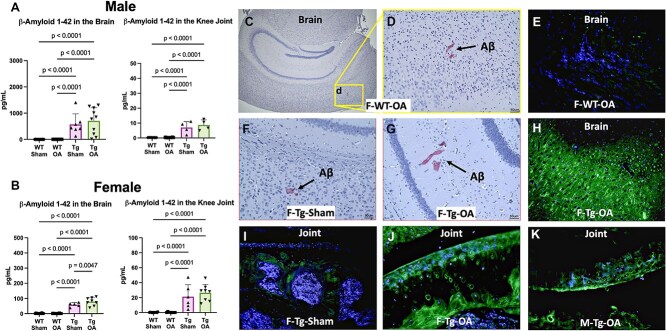
Amyloid levels in the brain and knee joint. Both male (A) and female (B) APP/PS1-Tg mice showed increased β-amyloid in both brain and in knee joint compared to WT mice by ELISA quantification of Aβ42 levels. While amyloid plaques were nearly undetectable in the brain of WT injured females via cargo red staining (C and D, red) or Thioflavin S staining (E, fluorescent green), the plaques were more evident in the brains of APP/PS1-Tg injured females (G and H) relative to Sham injured females (F). Also Sham injured joints showed low levels of β-amyloid (I), while both female (J) and male (K) injured joints displayed elevated levels of fluorescent Thioflavin S staining (J and K, green).

**Figure 4:**
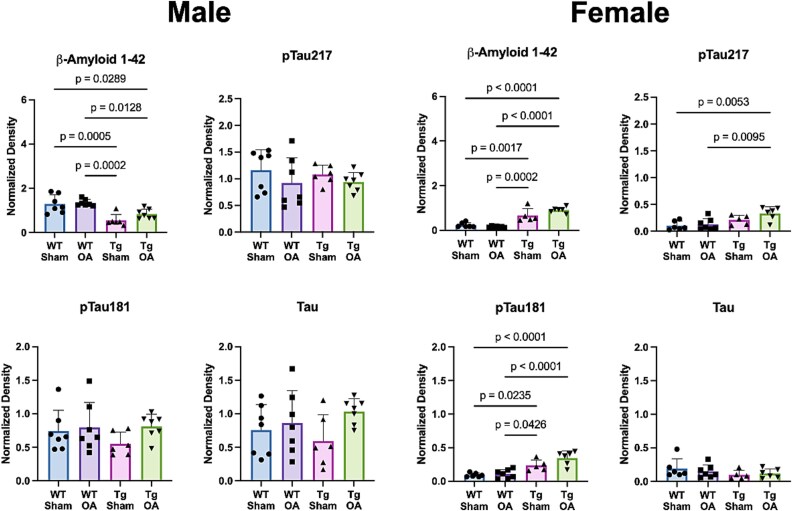
Western blot analysis of β-amyloid and tau in the brain. In male mice, WT mice showed greater β-amyloid 1-42 levels in the brain than APP/PS1-Tg mice in both the Sham and the OA groups. In female mice, APP/PS1-Tg mice exhibited significantly greater β-amyloid, pTau-217, and pTau-181 than in WT mice, particularly in the Tg-OA group.

In female mice, β-amyloid was nearly undetectable using ELISA, Western blot, or Thioflavin S and Congo red staining in WT mice (Figures 3–4). In injured APP/PS1-Tg mice (APP/PS1-OA), β-amyloid was seen sparsely deposited in the brain (Figure 3F and G), but this was significantly greater than in the APP/PS1-Sham mice. Similarly, we found that both male and female APP/PS1-OA mice had increased total β-amyloid 1-42 levels in the brain measured by ELISA (Figure 3A and B); this was associated with higher levels of β-amyloid 1-42, p-Tau181, and p-Tau217 in female APP/PS1-Tg mice measured by Western blot (Figure 4), compared to female WT-Sham and WT-OA mice.

### The deposition of β-amyloid and activation of pTau in the knee joint was affected by ACL injury and differed by sex

In female APP/PS1-OA mice, β-amyloid was increased in the femoral subchondral regions; some amyloid plaques were also observed in the articular cartilage (Figure 3J) relative to sham injured females (Figure 3I). In male mice, APP/PS1-Tg mice had higher β-amyloid 1-42 levels in the knee joints measured by ELISA and Thioflavin S staining in the articular cartilage (Figure 3K).

### Female APP/PS1-Tg mice exhibited cognitive decline compared to WT mice

#### Open field

**Figure 5:**
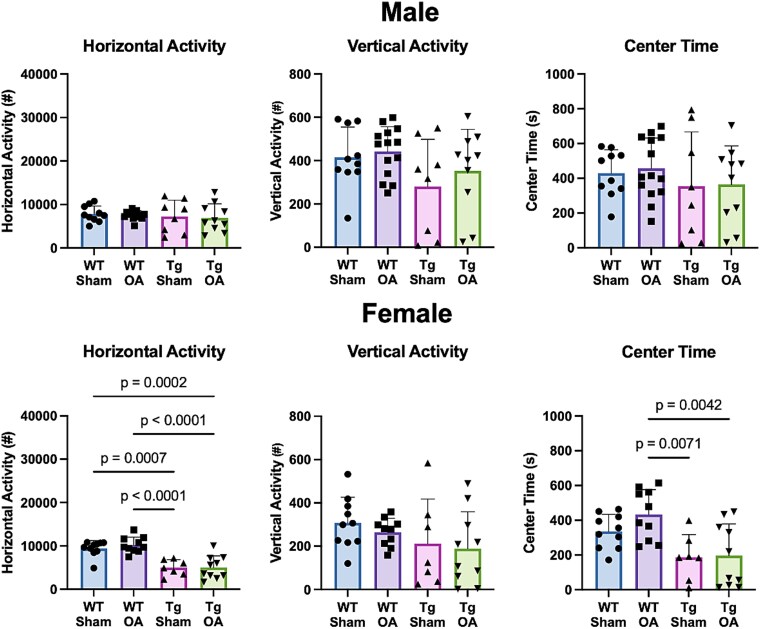
Analysis of exploratory behavior and voluntary movement using open field. Female APP/PS1-Tg mice exhibited decreased activity levels and exploratory behavior than in female WT mice, with significantly less horizontal movement and less time spent in the center of the open field. No differences in exploratory behavior were observed in male mice.

In female mice, we observed decreased activity levels and significantly less exploratory behavior in APP/PS1-Tg mice than in WT mice (Figure 5), but no significant differences were observed between OA and Sham mice. For male mice, there were no differences in exploratory behavior between APP/PS1-Tg mice and WT mice, and no changes were observed between OA and Sham mice.

#### Novel object recognition

**Figure 6:**
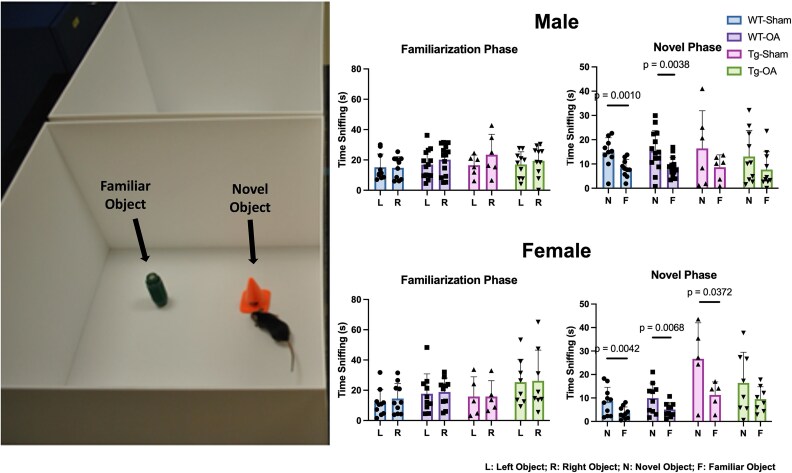
Novel object recognition. As expected, mice showed no preference for object location during the familiarization phase (L—left object, R—right object; both objects are novel during this phase). During the novel phase, all WT mice (both male and female) showed a significant preference for the novel object (N) compared to the familiar object (F). Female APP/PS1-Tg Sham mice also showed a significant preference for the novel object. However, female APP/PS1-OA mice did not show a significant preference for the novel object. No significant preference for the novel object was observed for either of the male Tg groups.

For both males and females, all mice showed no preference for object location during the familiarization phase. For female mice, both groups of WT mice and APP/PS1-Tg Sham mice showed a significant preference for the novel object during the novel phase. However, APP/PS1-OA mice did not have a significant preference for the novel object during the novel phase (Figure 6). For male mice, both WT groups show a significant preference for the novel object, but neither APP/PS1-Tg groups exhibited a significant preference for the novel object during the novel phase.

#### Spontaneous alternation

No significant differences were observed in the percentage of spontaneous alternation due to sex, genetic strain, or joint injury. The observed spontaneous alternation percentages were within the expected range (Figure S1).

#### Morris water maze

**Figure 7:**
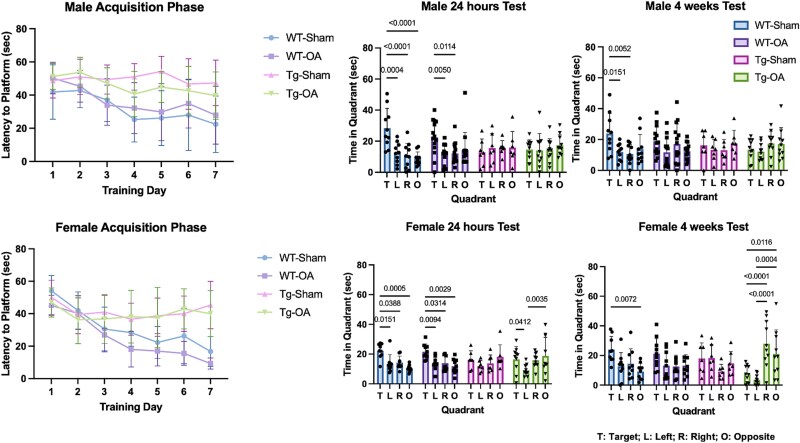
Morris water maze. During the acquisition phase, there were significant differences between WT and APP/PS1-Tg mice but no significant differences between Sham and OA mice. Both male and female WT mice showed decreasing latency time to locate the platform during the 7 d of the acquisition phase. However, neither male nor female APP/PS1-Tg mice showed significant decreases in latency time to the platform after 7 d of training. During the 24 h test, both male and female WT mice spent significantly more time in the target quadrant (T) compared to the other three quadrants (left—L, right—R, and opposite—O); neither male nor female APP/PS1-Tg mice spent more time in the target quadrant than the other 3 quadrants. Similarly, for the 4 wk test, both female and male WT-Sham mice spent more time in the target quadrant (T) than in the other quadrants (L, R, and O), but WT-OA and APP/PS1-Tg mice did not spend more time in the target quadrant.

Significant differences between WT and APP/PS1-Tg mice were observed during the acquisition phase and the testing phase of the Morris water maze test, though no significant differences were observed between Sham and OA mice (Figure 7). Both male and female WT mice learned the location of the platform during the acquisition phase, indicated by significantly lower latency time to find the platform after 7 d of training. In contrast, neither male nor female APP/PS1-Tg mice learned the location of the platform after 7 d of training. Both male and female WT mice passed the trial 24 h after last day of training, as shown by the mice spending significantly more time in the target quadrant compared to the other 3 quadrants during 60 s in pool with no platform present. Neither male nor female APP/PS1-Tg mice passed the 24 h trial; these mice did not spend more time in the target quadrant than the other 3 quadrants. All female WT mice and male WT-Sham mice passed the trial 4 wk after the last day of training. Male and female APP/PS1-Tg mice and male WT-OA mice did not pass the week 4 trial.

## Discussion

In this study, we examined how PTOA progression after ACL injury would affect AD-related pathologies and the progression of cognitive dysfunctions in a mouse model of AD (APP/PS1-Tg mice). We found that osteophyte formation, a key feature of OA, was more severe in APP/SP1-OA female mice than in WT-OA mice. Moreover, ELISA analysis (but not western blot) found that female APP/PS1-OA mice had higher β-amyloid 1-42 levels in the brain than WT-OA and APP-PS1-Sham mice. Female APP/PS1-OA mice also showed some signs of decline in cognitive function compared to APP/PS1-Sham mice and WT mice in the novel object recognition test. On the other hand, no significant differences in overall OA severity, β-amyloid levels in both the brain and joints, pTau formation, and cognitive functions were observed for male APP/SP1-OA mice. These results suggest potential crosstalk between OA-associated inflammation and brain degeneration and cognitive functions, including key differences between males and females.

A study by Kyrkanides et al.^4^ was the first to demonstrate the correlation between OA and the exacerbation of neuroinflammation leading to the worsening of AD pathology in mice. This study found that induction of OA in APP/PS1; Col1-IL1βXAT compound transgenic mice exacerbated and accelerated the development of neuroinflammation and increased the size of amyloid plaques. Similarly, a study by Gupta et al. investigated the relationship between AD and OA using 5xFAD transgenic mice, another genetic mouse model of AD, to determine whether OA initiated by destabilization of the medial meniscus (DMM) affected dementia progression and neuroinflammation.^5^ They found that OA in 5xFAD transgenic mice increased inflammatory cytokine levels in the brain and increased Aβ deposition and neuronal loss compared to Sham controls. These studies support systemic inflammation associated with OA as a mechanism driving neuroinflammation and cognitive decline in AD. Our study further supports this mechanism and adds novel insights with the use of a genetic mouse model of AD combined with a clinically relevant non-invasive ACL rupture model of PTOA along with additional neurocognitive tests.

The vast majority of people with OA are older than 45 yr, and women are more commonly affected than men^39^ and tend to present at more advanced stages of OA compared to men.^40^ The risk of OA increases in post-menopausal women,^41^ suggesting a link between OA and declining estrogen status. Estrogen deficiency is also speculated to be associated with cognitive impairment.^42^ Women with low circulating estradiol levels are nearly twice as likely to develop OA or experience joint pain and are at significantly higher risk of cognitive impairment and dementia, after adjusting for age, injury history, and BMI.^43^ Estrogen deficiency also results in increased pro-inflammatory cytokines,^44^ and long-term estrogen replacement therapy may provide a moderate level of protection against knee OA.^4^ It is possible that these hormonal associations with OA and cognitive impairment may relate to the observations in the current study of differences by sex of the mice in the association between OA and decline in cognitive function.

This study has some notable strengths including that this is one of the first studies to directly investigate a mechanistic relationship between OA and AD using clinically relevant mouse models (APP/PS1-Tg mice, non-invasive ACL rupture). We performed a comprehensive analysis of PTOA, evaluation of expression of pTau and Aβ in multiple tissues, and robust evaluation of neurocognitive function. The current study also reports analysis of sex differences for all outcomes.

There are also some limitations of this study that must be acknowledged. For example, we examined only one age of mice and only one time point post-injury. Additionally, APP/PS1-Tg mice are only one of several available mouse models of AD; it is possible that other genetic models would provide additional insight. Second, although the osteophyte scoring data presented in Figure 1 provide evidence that female APP/PS1-OA mice exhibited a modest (but statistically significant) increase in osteophyte score compared to WT-OA females, Figure 2 shows that very little mineralized osteophyte volume was observed in female WT-OA mice. This was an unexpected finding that is notably different than what we observed in our previous study of OA following non-invasive ACL injury in female mice.^45^ This difference may be due to using a different genetic strain of mice (non-Tg littermates vs purchased C57BL/6J mice) or using a different age of mice (7-mo-old vs 3-mo-old). Additional studies would be required to further interrogate this disparity. Third, although systemic inflammation associated with OA is hypothesized to be the mechanism affecting AD progression, this study did not specifically quantify systemic inflammation in these mice. Finally, group sizes may not have been sufficient to provide the statistical power for detecting sex- and OA-related differences in outcomes, especially for the behavior tests, which typically have large within-group variation.

### Conclusions

In summary, we found that OA in mice was associated with increased Aβ and pTau in the brain and joints, particularly in the brains of female APP/PS1-OA mice, and that female APP/PS1-OA mice exhibited increased osteophyte formation compared to WT-OA mice. Knee OA was also associated with beta-amyloid plaque deposition in the brain and some early signs of neurodegeneration in female AD mice, suggesting that OA may be a risk factor for the early onset of cognitive decline in AD. Given that AD is the most common form of dementia and shares similar demographic and potential underlying mechanistic elements with OA, both being age-related and low-grade inflammatory-associated diseases, this study supports the possibility that OA may represent a unique risk factor for AD.

## Supplementary Material

Figure_S1_ziag085
