# Supplementary figures and images for: Brain and knee joint degeneration following anterior cruciate ligament injury in a mouse model of Alzheimer’s disease
Source: JBMR Plus. 2026 May 7;10(7):ziag085. doi: 10.1093/jbmrpl/ziag085 (PMC13232624; doi:10.1093/jbmrpl/ziag085)

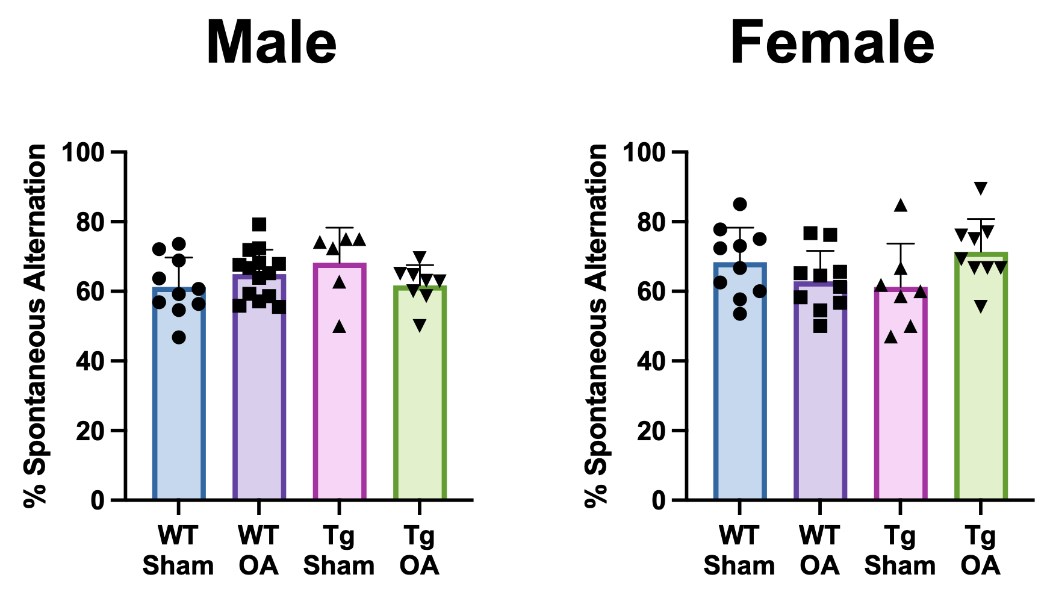

Supplement: Figure_S1_ziag085 [file figure_s1_ziag085.jpeg]
